# Myocardial Infarction as a Rare Cause of Otalgia

**DOI:** 10.1155/2014/106938

**Published:** 2014-11-13

**Authors:** Riza Dundar, Erkan Kulduk, Fatih Kemal Soy, Ersin Sengul, Faruk Ertas

**Affiliations:** ^1^Department of Otolaryngology, Harran University Medical Faculty, 63100 Sanliurfa, Turkey; ^2^Department of Otolaryngology, Mardin Government Hospital, 47000 Mardin, Turkey; ^3^Department of Otolaryngology, Nusaybin Government Hospital, 47000 Mardin, Turkey; ^4^Department of Cardiology, Dicle University Medical Faculty, 21000 Diyarbakir, Turkey

## Abstract

*Aim*. To present a case referred to our clinic with severe right ear pain but without any abnormal finding during otological examination and diagnosed as myocardial infarction and also to draw attention to otalgia which can occur secondary to myocardial infarction. *Case Report*. An 87-year-old female admitted with right ear pain lasting for nearly 12 hours and sweating on the head and neck region. On otolaryngologic examination, any pathological finding was not encountered. Her electrocardiogram revealed findings consistent with myocardial infarction. Her troponin values were 0.175 ng/L at 1 hour, and 0.574 ng/L at 3 hours. The patient was diagnosed as non-ST MI, and her required initial therapies were performed. On cardiac angiography, very severe coronary artery stenosis was detected, and surgical treatment was recommended for the patient. The patient who rejected surgical treatment was discharged with prescription of medical treatment. *Conclusion*. Especially in elderly patients with complaints of ear pain but without any abnormal finding on otoscopic examination, cardiac pathologies should be conceived.

## 1. Introduction

Otalgia seen after ear pathologies is termed as primary otalgia. However, primary otalgia can be frequently observed in other abnormalities. This symptom is termed as secondary or referred otalgia [1].

Sensory innervation of the ear originates from the V, VII, IX, and X cranial nerve and C2-C3 spinal nerves. Any abnormality which might occur in this neural net can lead to referred otalgia [2]. Complaint of secondary otalgia which can be seen in a wide spectrum of diseases is a frequently encountered symptom in temporomandibular joint abnormalities, dental diseases, pharyngotonsillitis, pathologies involving insertion site of the sternocleidomastoid muscle to the mastoid muscle, masseter muscle spasms, and arthritis of the cervical vertebra [3]. Otalgia secondary to myocardial infarction which we also saw in our case has been very rarely reported in the literature; however this important potentially life-threatening etiological factor should be always kept in mind [4, 5].

Myocardial infarction (MI) seen following atherothrombosis frequently presents with feeling of pressure on the chest, dyspnea, pain radiating to the arm, and neck or epigastric pain. Though it very rarely presents with otalgia, it is still seldom seen with only otalgia without accompanying classical symptoms [6–9].

In the present paper, we aimed to both present a case that was referred to our clinic with severe right ear pain but without any abnormal finding during otological examination and diagnosed as MI and also draw attention to otalgia which can occur secondary to MI.

## 2. Case Report

**Figure 1:**
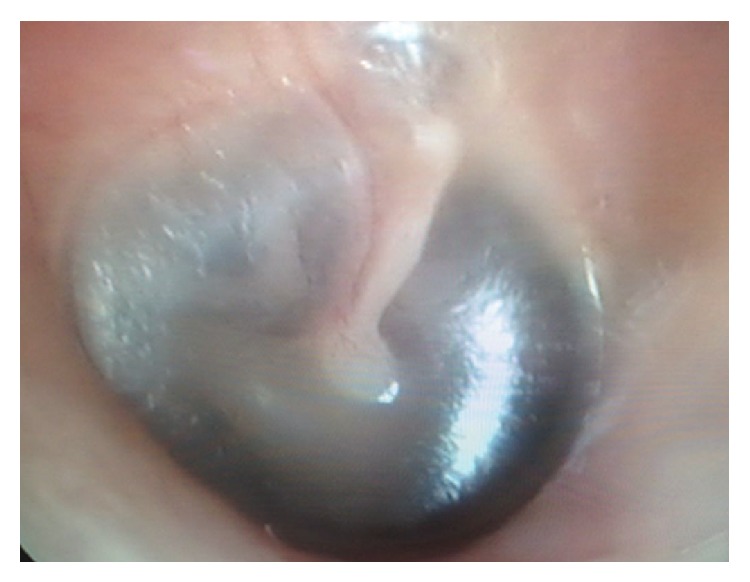
Otoscopic examination of the patient.

**Figure 2:**
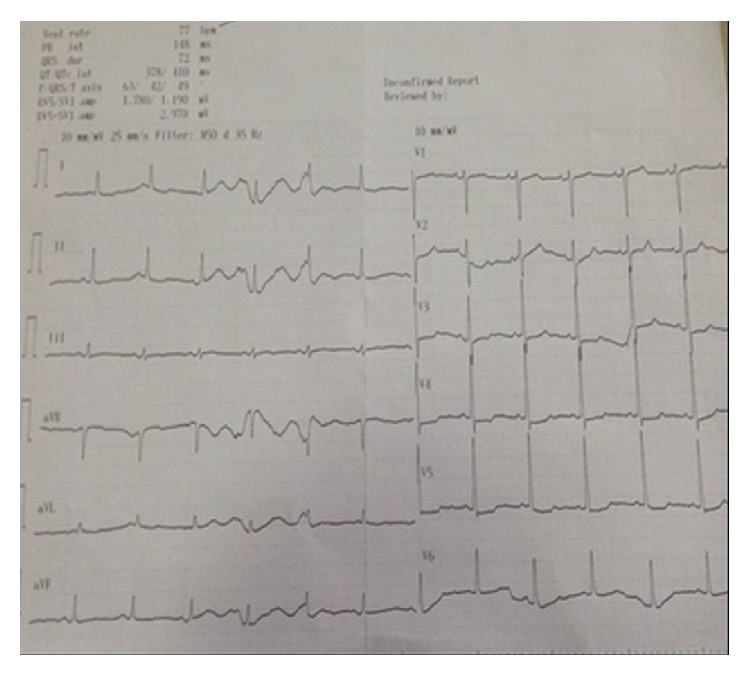
Electrocardiogram revealed findings consistent with myocardial infarction.

An 87-year-old female patient consulted ENT clinic with right ear pain lasting for nearly 12 hours and sweating on the head and neck region. On ENT examination of the patient any pathological finding was not encountered (Figure 1). The patient also indicated that she was receiving treatment for type 2 diabetes mellitus (DM), and she was referred to an internal medicine specialist for systemic examination. Her pulse rate was 98 bpm, and her arterial BP was measured as 130/100 mm Hg. Her electrocardiogram revealed findings consistent with MI (Figure 2). Her troponin values were 0.175 ng/L at 1 hour and 0.574 ng/L at 3 hours. Her routine biochemical and hemogram values were unremarkable. The patient was diagnosed as non-STEMI, and her required initial therapies were performed. Then she was referred to an advanced center for testing and treatment.

**Figure 3:**
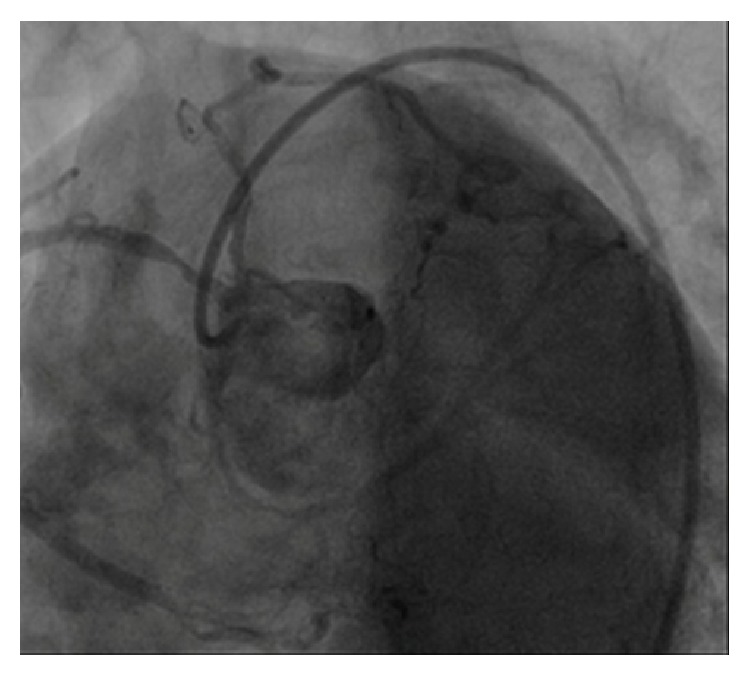
Severe coronary artery stenosis detected on cardiac angiography.

**Figure 4:**
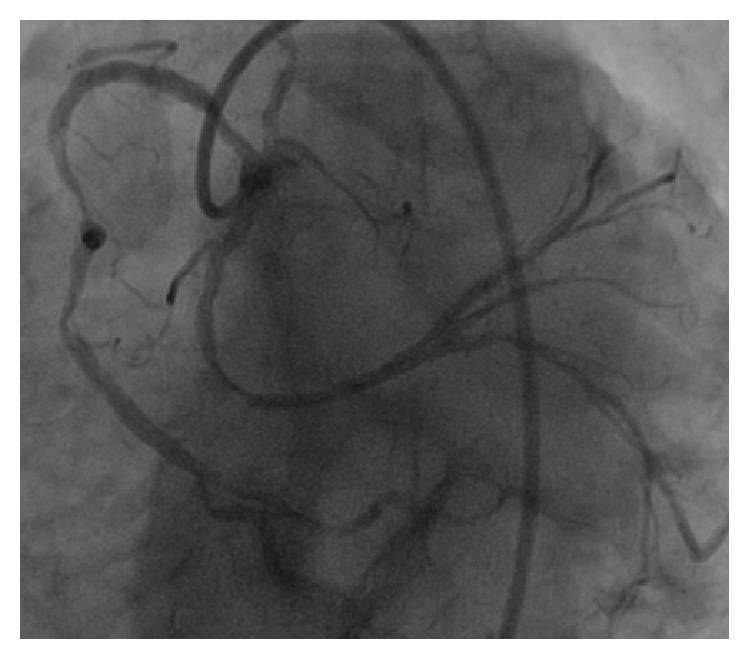
Severe coronary artery stenosis detected on cardiac angiography.

On cardiac angiography performed in an advanced center, very severe coronary artery stenosis was detected (Figures 3 and 4), and surgical treatment was recommended for the patient. The patient who rejected surgical treatment was discharged with prescription of medical treatment.

## 3. Discussion

Secondary otalgia is sensed by the ear but originates from a nonotologic source and poses a difficult diagnostic challenge to even the most experienced otolaryngologist. A negative otologic exam and persistent otalgia should suggest the possibility of referred otalgia. The neuroanatomic basis of referred otalgia rests within one of five general neural pathways listed above. One of these general pathways is via the glossopharyngeal nerve (cranial nerve IX) [10].

Jacobson's nerve, a derivative of the glossopharyngeal nerve, joins the caroticotympanic branches from the sympathetic plexus to form the tympanic plexus. This plexus provides sensation to the middle ear, upper eustachian tube, and medial surface of the tympanic membrane. Referred otalgia transmitted by the glossopharyngeal nerve may be secondary to lesions and/or inflammatory processes of the nasopharynx, palatine tonsil, soft palate, or posterior one-third of the tongue. Acute pharyngitis, tonsillitis, peritonsillitis, and peritonsillar abscess are common diseases that may cause secondary otalgia [11].

In a study performed by Taziki and Behnampour, secondary otalgia was most frequently caused by toothaches (62.8%), followed by pharyngeal infections (24.5%), and temporomandibular joint pathologies (8.5%) [12]. Kiakojoori and Tavakoli indicated that 45% of the patients with complaints of toothache had been referred to the hospital with complaints of otalgia [13]. In a study by Behnoud and Zandi, the authors reported temporomandibular joint pathologies as the most frequently detected etiologic factor for otalgia [14]. In a study by Kiakojoori and Tavakoli detected dental problems in 50% of the patients presented with otalgia [13]. Besides, Kim et al. published a case of Bell's palsy in a patient who consulted with complaints of otalgia whose physical examination did not reveal any other pathology [15].

The underlying pathophysiologic cause of secondary otalgia can be explained as autonomic dysfunction. In cases where this autonomic dysfunction involves the branches of the 10 cranial nerves (n. vagus), secondary otalgia may occur. Especially on areas innervated by nervus vagus auricular branch, this condition is more conspicuous. Referred otalgia can occur generally secondary to head and neck pathologies, but it can be rarely related to other pathologies. Pathophysiology of secondary otalgia detected in our patient has been explained with other mechanisms. According to a theory, sinoatrial node is innervated by right vagal nerve and perfused with right coronary artery. Right coronary artery occlusion leads to destructive changes in parasympathetic fibers of the vagal nerve with resultant secondary otalgia [6–9].

Our patient presented with complaints of severe ear pain and sweating on the neck region. Otoscopic and systemic examination of the patient who described right ear pain were unremarkable and systematic tests performed revealed the presence of MI.

As far as we know, otalgia which leads to acute arterial occlusion and coronary artery disease has been rarely reported in the literature. We could find only two case reports related to this subject in the literature [4, 5]. One of these cases was not diagnosed correctly, and the patient was diagnosed as primary ear pain at baseline and received long-term therapy accordingly. In conclusion, clinicians should not forget that ear and throat pain can be induced by cardiac ischemia, and one should be careful about outcomes with potentially life-threatening consequences.

## 4. Conclusion

Especially in elderly patients with complaints of ear pain but without any abnormal finding on otoscopic examination, cardiac pathologies should be conceived, and the patient should undergo systemic examination. This approach can be an intervention aiming at searching for the cause of secondary otalgia or as is in our case it can be a life-saving intervention.
